# Methylphenidate Differentially Affects Intrinsic Functional Connectivity of the Salience Network in Adult ADHD Treatment Responders and Non-Responders

**DOI:** 10.3390/biology11091320

**Published:** 2022-09-06

**Authors:** Martin Ulrich, Katharina Heckel, Markus Kölle, Georg Grön

**Affiliations:** 1 Section Neuropsychology and Functional Imaging, Department of Psychiatry, Ulm University, 89075 Ulm, Germany; 2 Department of Psychiatry and Psychotherapy, Bonn University, 53127 Bonn, Germany

**Keywords:** rs-fMRI, functional connectivity, adult ADHD, methylphenidate, treatment response, machine learning, striatum

## Abstract

**Simple Summary:**

Methylphenidate is frequently used to treat attention-deficit/hyperactivity disorder (ADHD). It has been reported to correct (aberrant) connectivity between brain regions. However, previous neuroimaging research has not provided a coherent picture. One possible reason is that methylphenidate may not necessarily result in a satisfactory clinical treatment response of every patient. This suggests that neuroimaging research about methylphenidate’s mode of action could benefit from subdividing patients into groups of treatment “Responders” and “Non-Responders”. However, previous research has not applied this idea so far. Here, we used machine learning techniques to discern Responders from Non-Responders based on clinical symptoms. Fifty-three stimulant-naïve adult ADHD patients underwent functional magnetic resonance imaging to determine functional connectivity at rest. Measurements were repeated after 6 weeks’ treatment with methylphenidate. We found that, prior to treatment, a specific part of the so-called salience network was hypo-connected with the bilateral putamen in the patient sample as a whole, compared with 50 healthy controls. After methylphenidate treatment, this aberrant connectivity was restored to almost “normal” levels in Responders, but not in Non-Responders. Involvement of the putamen is reminiscent of early positron emission tomography findings, already suggesting modulation of dopaminergic brain regions for the successful treatment of ADHD with methylphenidate.

**Abstract:**

Positron emission tomography (PET) studies have shown involvement of the striatum when treating adult attention-deficit/hyperactivity disorder (ADHD) with methylphenidate (MPH). Results from resting-state functional magnetic resonance imaging (rs-fMRI) for the same issue were less unequivocal. Here, a new analytical framework was set up to investigate medication effects using seed-based rs-fMRI analysis to infer brain regions with alterations in intrinsic functional connectivity (IFC) corresponding with ADHD symptom reduction. In a within-subjects study design, 53 stimulant-naïve adult ADHD patients were investigated before and after 6 weeks of MPH treatment, using two major clinical symptom scales and rs-fMRI. The same data were acquired in a sample of 50 age- and sex-matched healthy controls at baseline. A consensual atlas provided seeds for five predefined major resting-state networks. In order to avoid biasing of medication effects due to putative treatment failure, the entire ADHD sample was first categorized into treatment Responders (N = 36) and Non-Responders (N = 17) using machine learning-based classification with the clinical scales as primary data. Imaging data revealed medication effects only in Responders. In that group, IFC of bilateral putamen changed significantly with medication and approached almost normal levels of IFC. Present results align well with results from previous PET studies, with seed-based rs-fMRI as an entirely different neuroimaging method.

## 1. Introduction

Attention-deficit/hyperactivity disorder (ADHD) is the most frequently diagnosed neurodevelopmental disorder in childhood, which can persist into adulthood [1], with a worldwide prevalence estimated at around 2.5% for adults [2]. The main characteristic clinical features are inattentive behavior and/or impulsiveness and hyperactivity, with possibly negative consequences for an individual’s lifetime.

Among the various medications, stimulants (amphetamines and methylphenidate (MPH)) have generally been recommended as first-line pharmacologic treatment [3]. In 1955, the U.S. Food and Drug Administration first approved methylphenidate for treatment of ADHD. Since then, knowledge about the neurobiological effects of ADHD stimulant medication (e.g., [4]) has increased strongly. However, less has been learned about the mode of action by which stimulants produce therapeutic improvement, that is, changes in symptom expression or severity [5]. For in vivo assessment of medication-induced changes, neuroimaging has become a widely used method, among which so-called resting-state functional magnetic resonance imaging (rs-fMRI) has received major attention in the last decade. This approach has been used to infer ADHD-related pathophysiology from patterns of “intrinsic functional connectivity” (IFC) across the whole brain that is different from healthy comparisons (for a recent review, see [6]). Similarly, rs-fMRI has also been used to infer treatment-induced differences by comparing indices of IFC before and after medication, and how they differ from or align with IFC patterns obtained from healthy controls. However, a recent systematic review [5] of medication effects on brain IFC in patients with ADHD concluded that, so far, data do not support a coherent mechanistic hypothesis of medication effects.

This is interesting because with positron emission tomography (PET) as another neuroimaging method it has already been shown in healthy controls that methylphenidate acts primarily as an inhibitor of the dopamine transporter (DAT) by increasing dopamine (DA) availability in the striatum, which was inferred from reductions in ligand binding potential (e.g., [^11^C]raclopride; for review, see [4]). Later, this effect was also demonstrated for adult patients with ADHD. Long-term MPH treatment increased striatal DA availability [7], and individual increases were correlated with individual decreases in symptom severity, indicating a medication-mediated clinical effect [8]. So far, however, involvement of the striatum (i.e., caudate nucleus and putamen) and pallidum has not consistently been reported in rs-fMRI studies performed in adult ADHD under stimulant medication [9,10,11]. Only the study by Yang and colleagues [10] with amphetamine reported involvement of the posterior putamen, which was part of two larger left and right insular clusters where IFC with seeds in left dorso-lateral prefrontal cortex and bilateral medial prefrontal cortex decreased after medication. Post-hoc comparisons with data from healthy controls using the same seeds showed that, before medication, IFCs for these regions were significantly higher relative to controls, while post medication significant group differences were absent. Despite these results, the notion that the striatum is a core brain region mediating effects of stimulant treatment, as suggested by PET studies, has not been confirmed by other studies using rs-fMRI to evaluate stimulant treatment effects in adult ADHD.

One major possible reason for the observed inconsistence of previous IFC findings is that none of the reviewed studies has accounted for the typical and frequent observation that not every patient will benefit from stimulant medication (possible reasons: appropriateness of stimulant medication, duration, adherence, diagnosis, etc.). Critically, absence or at least different individual extents of treatment outcomes might condition, bias, or even mask medication effects when inference is drawn from the group of treated subjects in its entirety. To overcome this shortcoming, the present study was conducted in which 53 adult ADHD patients underwent a pre-to-post medication within-subjects design. All patients were stimulant-naïve as adults when enrolled, had an estimation of IQ, and were rigorously screened for comorbidities during medical examination. Importantly, the entire group of ADHD patients was stratified into two subgroups of patients: so-called Responders (R), whose symptom expression changed back to almost normal levels, and so-called Non-Responders (NR) who showed reduced symptom expression after MPH treatment but did not change back to normality. The amount of treatment success was operationally defined as changes in clinical scores on two symptom scales. From these scales, eight different aggregates of subscales were computed that typically inform the clinician about the extent of treatment success. As “features”, these aggregate measures were subjected to different standard machine learning classification algorithms constituting a majority voting ensemble, and its outcome was used to differentiate between ADHD patients as Responders and Non-Responders.

Resting-state fMRI was performed both at participants’ first visit and after around 6 weeks’ treatment with methylphenidate. From an additional sample of 50 healthy matched controls, an rs-fMRI scan was obtained only once. Seed regions for determining whole-brain IFC were derived from the freely available Consensual Atlas of REsting-state Networks (CAREN; [12]). Aimed at facilitating reproducibility of resting-state networks (RSN), the CAREN atlas was the result of a quantitative comparison of the five major RSNs (default mode, salience, central executive, sensorimotor, and visual networks) across currently available brain functional atlases.

Before the analysis of treatment effects on functional connectivity, we first compared IFC maps between ADHD patients (as a whole) and healthy controls, in order to infer brain regions of altered functional connectivity, that is, “hyperconnectivity” and “hypoconnectivity” in ADHD relative to healthy controls. Brain regions derived from the group comparisons above then served as regions of interest for the evaluation of medication effects in Responders vs. Non-Responders.

Taking into account clinical experience, we hypothesized that a non-zero portion of Non-Responders would be identified by machine learning classification. Whether and where in the brain patients would demonstrate reliably enough abnormal IFC patterns relative to controls was not predicted in greater detail, because all five RSNs should be investigated for this issue, and a recent meta-analysis [6] precluded further localized and directional expectations in terms of hypo- and hyperconnectivity.

Due to ethical considerations, it was not possible to conduct a placebo-controlled trial for the present clinical patient sample. Therefore, the preparatory work of response classification and exploration of brain regions for initial group-different IFCs also served to set up the boundary conditions which, when fulfilled, would permit inference of a medication effect. Specifically, we predicted that this effect can be inferred for observations where normalization of IFC in the Responders subgroup is reflected by statistically reliable pre-to-post medication IFC changes in the direction to normality for brain regions that were initially different between all ADHD patients and healthy controls. Furthermore, these changes to normality in the Responders subgroup should be significantly greater than in the Non-Responders subgroup who, in turn, should not show any pre-to-post differences.

## 2. Materials and Methods

### 2.1. Study Samples

Fifty-three patients with ADHD were included in the study. All data stem from their visits to the ambulatory outpatient device of the Ulm Psychiatric Hospital, where they were seeking medical help due to subjective complaints. After medical examination, patients were informed about the research project and asked if they wish to participate, while it was made explicit to them that their voluntary decision would not have any impact on their patient status. Written informed consent was obtained from each participant prior to MR imaging, and all other data acquisitions and diagnostic self- and third-party assessment scales were handed out to every participant after obtaining written informed consent. Importantly, patients had not previously been on stimulant medication as adults (i.e., they were stimulant-naïve at baseline). During the first visit (M1), diagnosis of ADHD was performed in every participant using the Diagnostic Interview for ADHD in Adults 2.0 (DIVA 2.0), and participants completed the Conners’ Adult ADHD Rating Scales (CAARS) for self-assessment. During the second visit (M2), around 6 weeks from M1, the same set of questionnaires was to fill out again. Third-party assessment for the CAARS was provided by life partners, mother, father, or sibling. Always the same person also performed the third-party assessment at the second point in time (M2).

In addition to the sample of ADHD patients, 50 healthy controls participated in the study, who were invited to the laboratory by oral advertisements and social media. Given the necessity to reach an average matching of controls along the dimensions age, sex, and years of school, this information was obtained from a first email or telephone contact during which participants were informed about the research project and asked whether they want to participate given that their demographic status matched the necessary prerequisites. After appearance at the laboratory, written informed consent was obtained, and the CAARS for self- and third-party assessment were handed out. Afterwards, the DIVA interview was performed by an experienced clinical psychologist (KH) who also conducted a semi-standardized interview to ask for present somatic, neurological, and/or psychiatric disorders, which had been defined as an exclusion criterion for controls. None of the healthy controls took any psychotropic medication or misused drugs of any kind. 

Written informed consent was obtained from each participant. The entire research project had been approved by the institutional review board (EA 196/15) and was in concordance with the Declaration of Helsinki. Data were acquired between October 2015 and October 2019.

### 2.2. Medication: Timeline and Dosing

Medication with methylphenidate followed typical clinical prescription according to German S3 guidelines. Patients were instructed to start treatment with methylphenidate retard 10 mg/day and to increase the daily dose by 10 mg per week up to a possible maximum dose of 60 mg/day, depending on individual efficacy and tolerability. In fact, patients achieved daily doses between 20 and 50 mg/day within the 6 weeks of treatment (mean duration: 47 days; SD: 7.7 days; and range: 42–70 days). Around 63% of the patients could be measured exactly after 42 days of regular intake. At their second visit (M2), patients did not take a sustained-release preparation before the measurement, but an unretarded dose of methylphenidate, equivalent to half the morning dose of the sustained-release preparation. The time of ingestion was 60–90 min before the rs-fMRI scan. The objective was a controlled ingestion on the day of measurement with a rapid onset of action and a single peak of methylphenidate action, beginning approximately 60–90 min after ingestion.

### 2.3. Blood Sampling and Analysis

In order to measure individual levels of methylphenidate, blood serum samples were taken immediately after rs-fMRI. Samples were frozen and sent to a local laboratory to measure levels of MPH in the blood serum and its first major, inactive metabolite ritalinic acid. For around 21% of the patient sample, levels of MPH were below the limit of detection. By contrast, the level of ritalinic acid was above the detection limit in each and every patient.

### 2.4. Magnetic Resonance Imaging

Resting-state fMRI was performed on a 3T Siemens MAGNETOM Prisma equipped with a 64 channels head coil. An echo-planar imaging (EPI) pulse sequence was used to acquire a series of 180 volumes during 6.54 minutes’ time. Scanning parameters: TR = 2400 ms; TE = 36 ms; bandwidth = 2004 Hz/pixel; PAT factor = 2 (GRAPPA mode); FOV = 220 mm; matrix size = 96 × 96; in-plane voxel size = 2.3 mm × 2.3 mm; number of slices: 36; slice orientation: AC-PC oriented; acquisition: interleaved; slice thickness: 3.6 mm; no gap; and flip angle: 78°. Phase encoding was in the anterior to posterior direction (A→P).

The risk of head motion was minimized by various foam pads that were applied within the already rather tight 64 channels coil. Subjects were instructed to lay still and to keep their eyes open and fixed on a fixation cross presented on an MR-compatible 32″ LCD screen (NordicNeuroLab AS, Bergen, Norway) located at the rear end of the MR gantry. Visibility of the fixation cross was accomplished by means of a double-mirror projection mounted on the 64 channels MR coil.

For anatomical reference, a high resolution T1-weighted structural image was obtained by administering a 3D magnetization prepared rapid acquisition gradient echo sequence (MPRAGE) with the following parameters: TR = 2300 ms; TE = 2.98 ms; inversion time = 900 ms; flip angle = 9°; FOV = 256 mm; matrix size = 256 × 256; voxel volume = 1 mm^3^; slice orientation: sagittal; PAT factor = 2 (GRAPPA mode); and scan time = 5.21 min.

### 2.5. Classification of ADHD Patients into “Responders” and “Non-Responders”

First, separately for each of the eight CAARS/DIVA feature dimensions (see Table 1, Results section), the raw data from time point M1 were z-transformed across ADHD patients and control subjects. We then trained an ensemble of two support vector machines (SVM) and three k-nearest neighbor (KNN) classifiers to distinguish between categories of “healthy controls” and “patients” based on the features. SVMs and KNNs were chosen as classification models because they had been shown to outperform other techniques in a comparison of nine supervised classifiers [13]. The uneven number of five classifiers aimed at forcing a binary majority vote in any case.

For training of the SVMs and KNNs, we made use of MATLAB 2019b’s (The MathWorks Inc., Natick, MA, USA) “fitcsvm” and “fitcknn” functions, respectively. The two SVMs differed in their kernel function for which we used a linear kernel, and a Gaussian kernel. All other parameters were kept as default: Box constraint: 1; kernel scale: 1; polynomial kernel function order: 3; solver: Sequential Minimal Optimization (SMO); kernel offset: 0; cache size: 1000; nu: 0.5; and expected outlier fraction: 0. With regard to the KNN classifiers, the only difference lay in their distance function. We chose to use the Euclidean distance, the cosine distance, and the Chi square distance. Although the Euclidean distance is considered the most widely employed distance metric, there are some fields of application where the cosine distance may achieve better classification results (e.g., [14,15]). The Chi square distance was selected because it was recently shown to yield superior results compared with other distance functions when dealing with medical data sets, irrespective of data type [16]. Similar to the SVMs, all other KNN model parameters were MATLAB’s default settings except for the nearest neighbor search method which was always set to “exhaustive”: number of nearest neighbors to find: 1; distance weighting function: equal; tie-breaking algorithm: smallest index among tied groups; and include ties: false.

The trained classifiers were then fed with the patients’ M2 data after adjusting them with the settings acquired during normalizing the patients’/controls’ M1 data. If, for a given patient, the majority of classifiers predicted the label “healthy control”, the patient was classified as “Responder”, otherwise as “Non-Responder”.

### 2.6. Seed-Based Functional Connectivity Analysis

Resting-state fMRI data were analyzed with the CONN functional connectivity toolbox 19c ([17]; RRID:SCR_009550; www.nitrc.org/projects/conn, last accessed: 25 March 2020) in combination with Statistical Parametric Mapping (SPM) 12 (r7487; Welcome Department of Cognitive Neurology, London, UK), operating under MATLAB 2019b. As the data sets from ADHD patients and control subjects differed in the number of functional runs (M1 for controls, and M1 and M2 for patients), both groups were analyzed separately using two CONN projects with otherwise identical parameters. For more details on the following processing steps, please refer to Nieto-Castanon [18]. Raw data were first preprocessed using CONN’s default preprocessing pipeline for volume-based analyses, starting with functional realignment and unwarping, followed by slice-timing correction. In a third step, functional outlier scans were detected using CONN’s “intermediate settings”, the criteria of which were frame-wise displacements above 0.9 mm or changes in the global BOLD signal above 5 standard deviations. Next, functional data were simultaneously segmented (into grey matter, white matter, and cerebrospinal fluid) and normalized to Montreal Neurological Institute (MNI) space. This procedure, known as “direct segmentation and normalization”, was also applied to the T1 images. Functional data, now having a resolution of 2 mm × 2 mm × 2 mm, were smoothed using a Gaussian kernel with 8 mm full width at half maximum (FWHM).

To remove potential confounding effects from the preprocessed functional data, CONN’s default denoising pipeline (“anatomical component-based noise correction procedure“ (aCompCor)) was administered. Noise was estimated and regressed out from the BOLD signal extracted from white matter (10 components) and cerebrospinal fluid masks (5 components). Effects of estimated motion (12 components) and outlier scans were also removed before linearly detrending and temporally band-pass filtering (0.008–0.09 Hz) the signal.

After the denoising procedure, seed-to-voxel functional connectivity was computed. As seed regions, we used the CAREN atlas [12], subdivided into Automated Anatomical Labelling Atlas 3 (AAL3v1; [19]) regions, as kindly provided by Gaelle Doucet (www.researchgate.net/publication/334042115_Consensual_Atlas_of_REsting-state_Networks_CAREN; retrieved 31 August 2021). Thereby, the originally reported five CAREN networks disassembled into 211 seed regions, for which bivariate correlations were computed between each seed’s average time course and all other voxels in the brain. The resulting images containing the Fisher’s Z transformed Pearson correlation coefficients for each seed/subject/time point were then further analyzed in SPM12 outside of the CONN toolbox.

### 2.7. Analysis of Medication Effects on IFC

In order to statistically infer regions of interest for ensuing inference of medication effects, two-sample t-tests were computed comparing each of the 211 seed-based connectivity maps between healthy controls and the entire group of ADHD patients. Given the screening character, an intermediate level of above-chance between-group differences was defined before entering this analysis. This significance threshold was computed (G*Power; [20]) a priori and was based on actual sample sizes of 53 adult ADHD patients and 50 healthy controls. Power was set to 0.8 [21], and large effect sizes of group differences of around 0.8 were required. For a t-test for independent samples, significance was computed at *p* < 0.001 given these settings. This level was not further corrected for multiple comparisons, neither at the voxel nor at the cluster level. An extent threshold of k = 5 was added to avoid spurious results. Testing for significant hyperconnectivity (patients-controls) and hypoconnectivity (controls-patients) was performed at this statistical threshold. Again, there was no further adjustment for multiple testing at this stage of the overall analytic procedure due to the compound requirements explained below.

Within the group-difference maps, we then investigated whether changes in IFC before (M1) and after (M2) medication were significantly different between both subgroups of patients with ADHD; that is, whether initial (M1) hyper- or hypoconnectivity changed back to “normal” levels after medication (M2) in the Responders subgroup (but not, or to a minor degree, in Non-Responders). Corresponding Group-by-Time interaction contrasts were set up in a full-factorial model that encompassed the main factors Group (Responders, Non-Responders) and Time (M1, M2). As the pure interaction effect remains uninformative with respect to the question whether the change in IFC in Responders is statistically significant, an additional contrast was formulated to test for this issue. Therefore, inference of medication effects was bound to the testing of two hypotheses in conjunction. Consequently, for inference of significant voxels, SPM’s minimum t-statistic (Conjunction Global) was used in combination with a family wise error rate (FWE)-corrected statistical threshold of *p* < 0.05 at the voxel level, and adjusted for the search volume derived from the significant directed between-group difference (ADHD vs. healthy controls) from prior screening.

## 3. Results

### 3.1. Demographic and Clinical Characteristics of the Entire ADHD Group and Healthy Controls

Table 1 summarizes all demographic and clinical variables of interest. As expected, the portion of female patients with ADHD was lower than that of male patients. The distribution of sex was however not significantly different between groups (Chi^2^(d.f. = 1) = 0.70, *p* = 0.403). In addition, groups did not differ with respect to age, averaged number of school years, and estimated IQ. Average scorings on psychopathometric (sub)scales and associated summary scores differed significantly between groups (all t ≥ 7.19, *p* < 0.001, Cohen’s d ≥ 1.42). 

**Table 1:** Demographic and clinical characteristics of study participants at first visit (M1).

Variable	ADHD Group	Healthy Controls	t-Value (d.f. 101)	*p*-Value	Cohen’s d
N	53	50			
Female/Male	15/38	18/32			
Age	27.0 (5.5)	26.2 (5.3)	0.79	0.429	0.16
Years of school	10.7 (1.7)	10.9 (1.7)	−0.43	0.668	−0.09
Estimated IQ	113.2 (12.8)	116.2 (11.8)	−1.24	0.217	−0.24
DSM-IV A1	7.01 (1.25)	0.22 (0.51)	36.06	<0.001	7.11
DSM-IV A2	4.74 (2.49)	0.32 (0.77)	12.01	<0.001	2.37
CAARS_DSM-IA_S	19.11 (4.18)	5.24 (3.41)	18.40	<0.001	3.63
CAARS_DSM-HY/I_S	14.72 (5.89)	4.72 (3.29)	10.55	<0.001	2.08
CAARS_DSM-ADHD_S	33.60 (8.50)	9.96 (5.84)	16.36	<0.001	3.23
CAARS_DSM-IA_O	16.79 (5.07)	4.58 (3.55)	14.09	<0.001	2.78
CAARS_DSM-HY/I_O	12.23 (6.32)	4.70 (3.97)	7.19	<0.001	1.42
CAARS_DSM-ADHD_O	29.00 (9.33)	9.28 (6.56)	12.34	<0.001	2.43

Values are means; standard deviations (SD) in parentheses; IA: inattention; HY/I: hyperactivity/impulsivity; _S: self-assessment; _O: assessment by third party (others).

### 3.2. Classification of Responders and Non-Responders from Symptom Severity Scales

**Figure 1:**
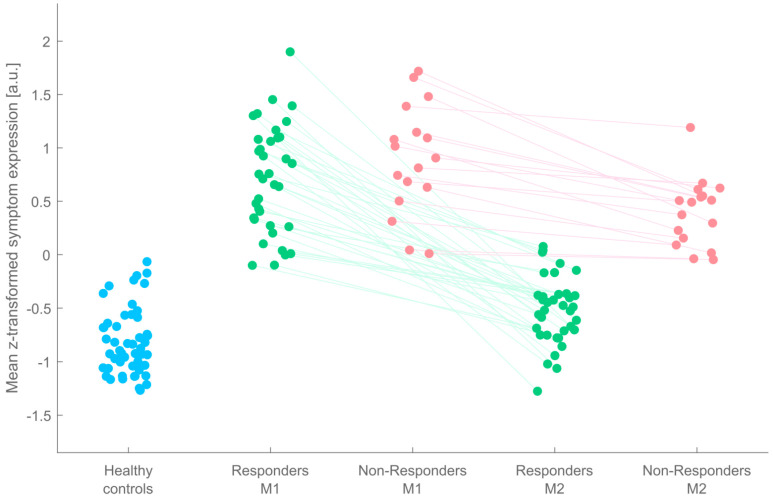
Graphical depiction of machine learning-based classification of adult patients with ADHD who changed back to almost normal levels of individual symptom expression (Responders, green) after 6 weeks of methylphenidate medication and those who did not (Non-Responders, red). The values represent individual mean z-transformed symptom expression, averaged across the 8 feature dimensions, before (M1) and after (M2) treatment with methylphenidate and for healthy controls (blue). Abbreviation: a.u.: arbitrary unit.

Based on the clinical data obtained from ADHD patients’ second visit (M2) after around 6 weeks of MPH treatment, the SVM/KNN majority voting ensemble classified 36 patients as treatment Responders, and 17 patients as Non-Responders (Figure 1).

As machine learning-based classification into subgroups of Responders and Non-Responders does not inform about how group differences are expressed in numerical clinical average rating scores and their changes from M1 to M2, Table S1 summarizes all demographic and clinical variables of interest for both groups separately. We also added classical variance-analytical results from Group-by-Time interaction effects, which permitted to calculate effect sizes for each interaction term.

### 3.3. Medication Effects on IFC

As outlined above, medication effects were inferred by testing for significant changes in IFC between the first visit and after around 6 weeks of medication with MPH. This difference was further stratified by the prerequisites that (a) the entire ADHD group had significantly different IFC parameters relative to healthy controls, (b) that the direction of pre-to-post-medication changes in IFC was opposite to the direction initially observed by the above contrast (i.e., hyperconnectivity in ADHD should reduce to “normal” levels, and vice versa), (c) that changes “back to normality” in the group of treatment Responders should be greater than that of Non-Responders (Group-by-Time interaction), and (d) that the IFC change in Responders should be significant per se, so that inference of a medication effect is not merely driven by the significant Group-by-Time interaction effect above (c).

**Figure 2:**
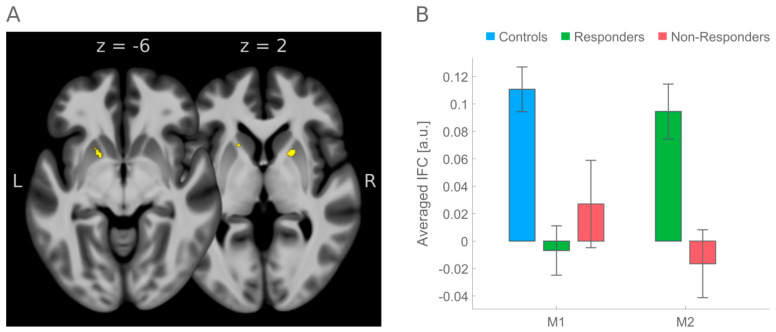
(**A**) Medication effect in left and right putamen superimposed on transversal slices from the mean 3D T1-weighted MPRAGE image in stereotactic MNI space, averaged across all participants (N = 103). For purposes of better visibility of the medication effect from the combined hypothesis testing, the resultant SPM was thresholded at a level of *p* < 0.001, uncorrected at the voxel level; exact statistics with family wise error rate correction at the voxel level are summarized in Table 2. Visualization of effects was performed with MRIcroGL [22]. The mean T1 image was computed with CAT12 (http://www.neuro.uni-jena.de/cat; last accessed: 21 March 2022); (**B**) Bar charts, created with Gramm [23], summarize mean and standard error of the mean (SEM) of IFC, averaged across all 17 voxels significant at the level of *p* < 0.05, FWE-corrected, as summarized in Table 2. Abbreviations: M1: pre-medication; M2: post-medication; a.u.: arbitrary unit; IFC: intrinsic functional connectivity; L: left; R: right.

Fulfillment of all prerequisites was observed for only one result pattern that was located in left and right putamen (Figure 2). According to the CAREN atlas, the seed for the corresponding rs-fMRI correlation maps was a subregion of the left precentral gyrus, forming part of the Salience RSN (see Figure S1 for a visualization of the seed region and aspects of the Salience RSN). At baseline, healthy controls demonstrated greater positive IFC with this seed than all ADHD patients in left putamen (peak voxel: x = −18; y = 6, z = −6; z-value = 4.33; *p* < 0.001; cluster size = 163 voxels) and right putamen (peak voxel: x = 20; y = 8, z = 4; z-value = 3.59; *p* < 0.001; cluster size = 36 voxels). It was only for these two regions that the compound requirements were fulfilled. Treatment Responders showed a significant increase in IFC from pre-to-post medication in the direction of healthy controls, which was also significantly greater in Responders than in Non-Responders (Group-by-Time interaction). The result of the latter combined hypothesis testing is summarized in Table 2.

## 4. Discussion

The present study used seed-based resting-state fMRI to investigate effects of methylphenidate treatment in adult patients with ADHD with pre-to-post-medication changes in IFC as the central dependent variable. Because these neuroimaging markers were treated as secondary informants about therapeutic success, the ADHD sample was first subdivided into groups of treatment Responders (N = 36) and Non-Responders (N = 17) so that insufficient clinical treatment responses would not bias analysis of neuroimaging data. Categorization was based on a majority vote obtained from an ensemble of five standard machine learning classifiers (SVM and KNN, with different settings) using typical data aggregations from two clinical rating scales (DIVA 2.0, and CAARS) as features. Five resting-state networks (default mode, salience, central executive, sensorimotor, and visual networks) with corresponding seeds were investigated. Seeds were taken from the Consensual Atlas of REsting-state Networks (CAREN; [12]). Inference of medication effects were constrained to regions where an initial screening had shown significant differences in IFC between the group of healthy controls (N = 50) and the entire group of ADHD patients (N = 53). Inference of medication effects was conditioned by testing for significant pre-to-post medication changes that were (a) in the direction of IFC in healthy controls, (b) were greater in treatment Responders than in Non-Responders, and (c) significantly increased from pre-to-post medication. These constraints led to observance of a significant medication effect on initial hypoconnectivity between a precentral seed region (belonging to the salience RSN) and left and right putamen.

Comparing previous rs-fMRI studies on MPH effects in adult ADHD patients with present results is difficult as those studies have either used a different treatment (amphetamine) [10], or aggregated across participants [11] treated with either amphetamine (N = 12) or methylphenidate (N = 7), in combination with a different rs-fMRI metric (community detection and node dissociation) and unknown history of stimulant medication [11], or focused on just one resting-state network (DMN) in men only [9], and did not consider data from healthy controls. In addition, common to all previous studies, study samples were markedly smaller than the present one ([10]: 16 ADHD, 21 healthy controls downloaded from 1000 Functional Connectomes Project; [11]: 19 ADHD, 31 healthy controls; [9]: 18 ADHD, no healthy controls). Moreover, none of the studies so far has followed the proposed multi-step stream of inference of medication effects, accounting for treatment Responders and Non-Responders. Nevertheless, if a comparison with previous results is to be made, among the studies referenced above, at least one [10] reported involvement of the putamen which was part of two larger left and right insular clusters that were, before medication, hyperconnected with left dorsolateral and bilateral medial prefrontal cortex in adult ADHD patients. After stimulant medication with amphetamine, this connectivity was reduced and aligned with the extent of connectivity observed in healthy controls; that is, the change from hyper- to almost normal connectivity pointed towards the opposite direction as reported here.

Better support for the validity of present results has been provided by studies [8,24,25] using [^11^C]raclopride PET imaging as a different imaging modality. Quantified changes in [^11^C]raclopride binding were interpreted that methylphenidate binds to the dopamine transporters, thereby enhancing extracellular dopamine in the basal ganglia, that is, caudate nucleus and particularly adjacent putamen, very similar to the results reported here (see Figure 2). This similarity is even more interesting because the seed for the actual brain region belongs to the so-called salience RSN. This aligns well with the saliency interpretation of MPH’s mode of action derived from those previous PET studies [8,24,25] because dopamine is a neurotransmitter well-known to signal the saliency of stimuli and to drive motivation for goal-directed behavior [26,27,28].

The present study has some limitations. Ethical considerations did not permit a placebo control in order to contrast IFC changes over time under treatment and no-treatment. We therefore cannot preclude that pre-to-post changes in IFC in the Responders group were also driven by unspecific changes over time. However, the observation that these changes clearly dissociated from the non-changes in the Non-Responders group, and that Responders’ IFC alterations in the direction of normality were evident in brain regions where previous PET imaging had already shown MPH treatment effects, renders the possibility less likely that present medication effects merely happened by chance.

While a major part of the present analysis stream (i.e., initial separation into Responders and Non-Responders, screening for dysfunctional IFC, and usage of a normative atlas for seed-based IFC calculation) would have been part of preregistering the study protocol including imaging and group analysis, it must be noted that this study was not preregistered. Present results are therefore exploratory and await empirical substantiation to support clinical relevance. This limitation relates not only to the inference of treatment effects but also to the inference of initial group differences between healthy controls and the entire ADHD group.

The prerequisite for inference of medication effects for only those brain regions where IFC was initially different between patients and controls can obscure observation of other brain regions bearing medication effects that may also mediate ADHD symptom reductions. Insofar, the strictness applied here can be acknowledged as a limitation. The inverted analytical sequence, with a focus first on medication effects in ADHD patients and then followed by post-hoc comparisons to infer how these effects relate to normal data, can be informative [10]. This, however, does not necessarily have to be the case, because post-hoc comparisons to control data can theoretically be of any direction then, in terms of hyper- or hypo- or non-different IFC, and interpretation of the direction of changes would rely on this outcome; that is, while the strictness applied here is helpful in avoiding interpretational ambiguity of post-hoc comparisons with control data, it is important to acknowledge that the unfolding of MPH’s mode of action is not necessarily confined to brain regions which are dysfunctional prior to medical treatment in the sense of a “defect repair mechanism”. Rather, MPH could also affect other brain loci that are initially inconspicuous, and further research is necessary to investigate this issue.

## 5. Conclusions

Despite these limitations, we conclude that differentiation between Responders and Non-Responders to MPH treatment appears as a helpful and fruitful approach to further investigate neuroimaging markers of medication effects in (adult) ADHD.

## Figures and Tables

**Table 2:** Medication effects inferred by combined hypothesis testing for significant Group-by-Time interaction in the presence of significant pre-to-post-medication increase in IFC in ADHD Responders.

Anatomical Region	x	y	z	z-Value	*p* (Voxel)	Cluster Extent	*p* (Cluster)	Cohen’s d
Putamen/Pallidum, left	−16	8	−2	4.01	0.007	12	0.009	0.70
Putamen/Pallidum, right	18	8	2	3.60	0.030	5	0.020	0.65

*p*-values are family wise error rate (FWE)-corrected for multiple comparisons, adjusted for the search volume obtained from the initial contrast of healthy controls minus all ADHD patients; x, y, z are MNI coordinates.

## Data Availability

Data cannot be shared publicly because of the European General Data Protection Regulation (GDPR). It was missed with the beginning of data acquisition in 2015 to obtain a formal written and signed consent from each participant, which regulates the transfer of biological material, an umbrella term, which also includes public sharing of imaging data.

## Supplementary Materials

The following supporting information can be downloaded at: https://www.mdpi.com/article/10.3390/biology11091320/s1, Table S1: Demographic and clinical characteristics of treatment Responders and Non-Responders of ADHD patients at baseline (M1) and after around 6 weeks of methylphenidate medication (M2); Figure S1: Visualization of aspects of the Salience Resting State Network and the seed region.
