# Supplementary material for: Methylphenidate Differentially Affects Intrinsic Functional Connectivity of the Salience Network in Adult ADHD Treatment Responders and Non-Responders
Source: Biology (Basel). 2022 Sep 6;11(9):1320. doi: 10.3390/biology11091320 (PMC9495306; doi:10.3390/biology11091320)
Supplement: Supplementary file 1 [file biology-11-01320-s001.zip › biology-1863260-supplementary.pdf]

Methylphenidate Differentially Affects Intrinsic Functional Connectivity of the Salience Network in Adult ADHD Treatment Responders and Non-Responders

Martin Ulrich [1,*,†], Katharina Heckel [1,†], Markus Kölle [2] and Georg Grön [1]

[1] Section Neuropsychology and Functional Imaging, Deptartment Psychiatry, Ulm University, 89075 Ulm, Germany

[2] Department of Psychiatry and Psychotherapy, Bonn University, 53127 Bonn, Germany

* Correspondence: martin.ulrich@uni-ulm.de

† These authors contributed equally to this work.

Supplementary Material

Table S1. Demographic and clinical characteristics of Responders and Non-Responders of ADHD patients at baseline (M1) and after around six weeks of methylphenidate medication (M2).

**Variable**	**Responders**		**Non-Responders**		**t-value (d.f. 51)**	**p-value**	
N	36		17				
Female/Male	11/25		4/13				
Age	27.3 (5.4)		26.4 (5.6)		0.61	0.546	
Years of school	10.9 (1.7)		10.4 (1.8)		0.90	0.375	
Estimated IQ	115 (11.7)		109 (14.4)		1.51	0.136	
	**M1**	**M2**	**M1**	**M2**	**Group-by-Time interaction F(1,51)**	**p-value**	**Cohen's d**
DSM-IV A1	6.86 (1.27)	0.64 (0.87)	7.47 (1.12)	4.53 (1.84)	43.08	< 0.001	1.84
DSM-IV A2	4.61 (2.26)	0.47 (0.81)	5.00 (2.98)	2.82 (2.16)	9.27	0.004	0.85
CAARS_DSM-IA_S	18.31 (3.95)	8.06 (4.14)	20.82 (4.25)	17.18 (3.94)	15.90	< 0.001	1.12
CAARS_DSM-HY/I_S	14.08 (5.42)	6.42 (3.32)	16.06 (6.76)	13.00 (5.36)	10.39	0.002	0.90
CAARS_DSM-ADHD_S	32.33 (7.33)	14.47 (6.31)	36.29 (10.29)	29.59 (6.89)	14.94	< 0.001	1.08
CAARS_DSM-IA_O	16.58 (5.20)	8.78 (4.29)	17.24 (4.91)	15.12 (4.12)	10.89	0.002	0.92
CAARS_DSM-HY/I_O	11.97 (6.14)	6.25 (3.32)	12.76 (6.84)	10.29 (4.97)	3.87	0.055	0.55
CAARS_DSM-ADHD_O	28.56 (9.54)	15.00 (6.27)	29.94 (9.09)	25.29 (6.73)	7.99	0.007	0.79

Values are means; standard deviations (SD) in parentheses; IA: inattention; HY/I: hyperactivity/impulsivity; _S: self-assessment, _O: assessment by third party (others)

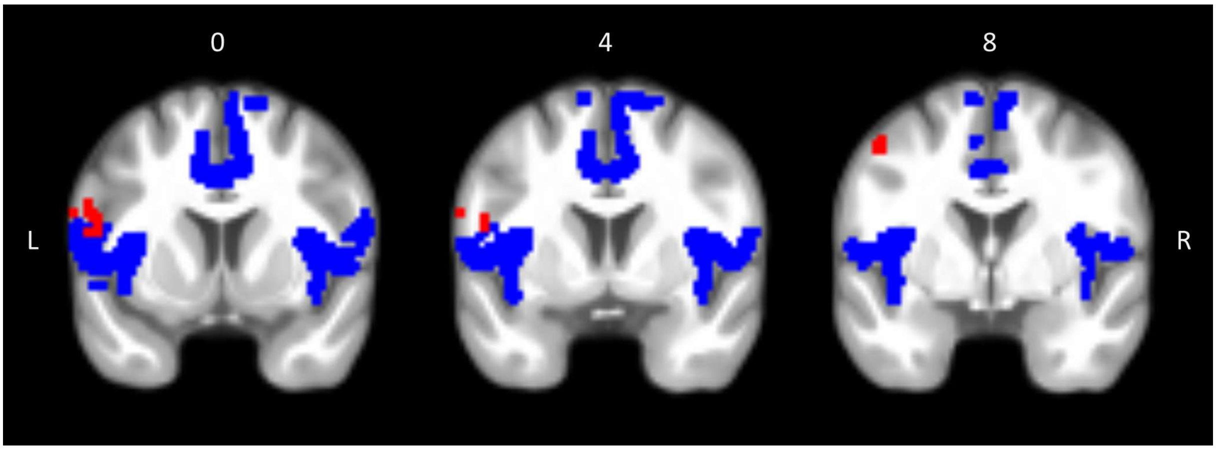

Figure S1. Visualization of aspects of the Salience Resting State Network (RSN) in blue and the seed region belonging to this RSN in red color. Please note that only those aspects of the Salience RSN are visualized where it coincided with the visualization of the seed region. A full picture of the Salience RSN can be found in: Doucet, G.E.; Lee, W.H.; Frangou, S. Evaluation of the spatial variability in the major resting-state networks across human brain functional atlases. *Hum. Brain Mapp.* 2019, *40*, 4577-4587. Coordinates refer to MNI space. Abbreviations: L: left; R: right.
